# Stereotactic Radiation Therapy (SRT) for Brain Metastases of Multiple Primary Tumors: A Single Institution Retrospective Analysis

**DOI:** 10.3389/fonc.2019.01352

**Published:** 2019-12-10

**Authors:** Lei Gu, Shuiwang Qing, Xiaofei Zhu, Xiaoping Ju, Yangsen Cao, Zhen Jia, Yuxin Shen, Fei Cao, Fang Fang, Huojun Zhang

**Affiliations:** Department of Radiation Oncology, Changhai Hospital Affiliated to Second Military Medical University, Shanghai, China

**Keywords:** stereotactic radiation therapy, brain metastasis, overall survival, prognostic factors, non–small-cell lung cancer

## Abstract

**Purpose:** To evaluate the efficiency and side effects of stereotactic radiation therapy (SRT) with or without other treatments for brain metastases (BM) from various primary tumors.

**Methods:** This was a retrospective analysis of 161 patients with brain metastases treated with SRT. Clinical data, EGFR mutation status and survival data were collected. Follow-up data was analyzed until December 2018. Kaplan-Meier and Cox proportional hazards regression analyses were used for the survival analysis.

**Results:** The median overall survival (OS) was 19 months. No difference was observed in OS between SRT group and SRT + whole brain radiation therapy (WBRT) groups (*p* = 0.717). Statistically significant factors of better OS after univariable analysis were no extracranial metastases (*p* = 0.016), BED_10_-SRT≥50Gy (*p* = 0.049), oligometastases (1–3 brain metastases) (*p* < 0.001), GPA score≥2.5 (*p* = 0.003), RPA class I (*p* = 0.026), NSCLC tumor type (*p* = 0.006), targeted therapy (*p* < 0.001) and controlled extracranial disease (*p* = 0.011). Multivariate analysis indicated that higher BED_10_-SRT (≥50Gy, HR = 0.504, *p* = 0.027), controlled extracranial disease (HR = 0.658, *p* = 0.039) and targeted therapy (HR = 0.157, <0.001) were independent favorable predictors for OS. Besides that, we also find that the median overall survival (OS) was 22 months in NSCLC patients and controlled extracranial disease (HR = 0.512, *p* = 0.012) and targeted therapy (HR = 0.168, < 0.001) were independent favorable predictors for OS.

**Conclusion:** For patients with brain metastases, stable extracranial disease, higher BED_10_-SRT (≥50Gy) and targeted therapy may predict a favorable prognosis.

## Introduction

Brain metastases are the most common intracranial malignancies, about 10–30% cancer patients develop brain metastases during the course of their diseases (1, 2) and 20 to 30% of patients with BM die as a result of poor local control (3). BM is one of the main causes seriously reduces the patients' life quality (4). Almost 40% patients will develop brain metastases during the course of their disease in non–small-cell lung cancer (NSCLC), and it may be even higher in those patients with epidermal growth factor receptor (EGFR) mutation (4, 5). Patients with EGFR-mutation may have a greater proportion of being diagnosed with brain metastases because of longer survival owing to targeted therapy and Central Nervous System (CNS) imaging technique improvement (6, 7).

There are various approaches for the treatments of brain metastases including surgical resection, stereotactic radiosurgery (SRS), whole brain radiation therapy (WBRT), systemic steroids and other combinations. Mintz et al. (8) demonstrated that surgery followed by WBRT obtained longer overall survival and better response to treatment compared to WBRT alone; but no differences were found in recurrence rate in metastasis site. Similar results were also published by Mintz et al. (8) Patchell et al. (9), and Vecht et al. (10).

In the RTOG 9508 trial (11), 333 patients with 1–3 brain metastases were randomly assigned to either WBRT or SRT-WBRT. WBRT and stereotactic boost treatment improved functional autonomy (KPS) for all patients and survival for patients with a single unresectable metastasis. In the secondary analysis performed after 10 years (12), 252 patients have been rearranged according to the GPA score. Survival advantage was found only in patients with higher GPA score (3.5–4) no matter the numbers of brain metastases.

## Methods and Materials

### Patient Selection

From February 2012 to June 2017, 161 patients with single or multiple (up to 7) brain metastases with good performance status and synchronous/metachronous primary tumor were treated at the Radiation Therapy Department, Changhai Hospital, Naval Medical University. Follow-up data was analyzed until December 2018. The study was approved by the independent Ethics Committee of our hospital and all patients signed informed consents. Data necessary for analysis were extracted, compiled, and verified against patients' archived medical records. Data analyzed included primary cancer, karnofsky performance score (KPS), Graded Prognostic Assessment (GPA) score, recursive partitioning analysis (RPA) classification at the time of SRT, site of intracranial metastases, number of lesions treated, present of extracranial metastases, and date of death or last follow-up, SRT treatment records, WBRT treatment records, and status of primary disease and systemic disease at SRT.

## Radiation Treatment Technique

WBRT treatments were administered with 21EX Linear Accelerator (Varian Medical Systems, Palo Alto, CA) using 3D-CRT. SRT were delivered with CyberKnife robotic radiosurgery system (Accuray, Sunnyvale, USA) Metastases were diagnosed based on contrast enhancement MRI imaging. The contours were delineated and reviewed by attending radiation oncologists. Gross tumor volume (GTV) was defined as the area of contrast enhancement on T1-weighted MRI images. The dose was prescribed to a 75% (at least) isodose. The precise prescription varied with tumor volume, site, and neurologic symptoms.

## Patients' Follow Up

Patients were followed up at regular intervals (every 3 month within 1 year, every 6 month 1 year later) to determine tumor status and the presence of symptoms. All data (clinical, radiological, therapeutic options and response to treatment) were collected by two physicians and the accuracy of the data were confirmed by two administrators. Toxicities were scored according to the Common Toxicity Criteria Adverse Events version 4 (CTCAE v.4). Acute toxicity was defined within 3 months following treatment. Toxicities were graded per RTOG acute central nervous system (CNS) morbidity scoring criteria. Acute toxicity outcomes included patient reported fatigue, headache, nausea/vomiting, dizziness/imbalance, motor neuropathy, sensory neuropathy, edema, neurocognitive dysfunction, and seizures.

## Formulas and Statistics

The biological effective dose (BED) was calculated for every metastasis treated according to the following formula, where n is the number of fractions and d is the dose per fraction. Following the Linear quadratic model, a value of 10 was used for the α/β-ratio. BED = nd^*^[1+d/(α/β)]. OS started with the first day of irradiation and was estimated using Kaplan–Meier analysis. Subgroups were compared using the log-rank test for univariate analysis and the Cox proportional hazard model for multivariable analysis. A *p* < 0.05 was considered statistically significant. A *p* < 0.1 was considered a trend and was the criterion for inclusion in multivariable analysis. All statistical analyses were performed using IBM SPSS Statistics 19 (New York, USA).

Patient characteristics were presented with descriptive statistics. Overall survival (OS) curves were calculated by the Kaplan–Meier method. Median OS and 95% confidence intervals (CIs) were reported. To identify potential predictive factors of OS, a univariate analysis was done with Cox proportional hazards regression within the training cohort. Factors with a *p* < 0.05 in the univariate analysis were entered as candidate variables into a multivariate stepwise Cox regression model (conditional backward selection).

## Results

### Patient Clinical and Treatment Characteristics (Table 1)

One Hundred and sixty-one patients with 305 brain metastases treated with SRT between February 2012 and June 2017 were enrolled in the study. The number of lesions ranged from 1 to 7 (median number of metastases was one). Most Patients (88.2%) had the KPS of 70 or higher. The majority of the patients were male (64%) and the median age was 61 years (range, 33–87). 32.9% patients showed synchronous brain metastases. 56.5% patients showed extracranial metastases. Ninety-three patients (57.8%) had systemic therapies including chemotherapy or targeted therapy. The patients demonstrated a range of primary malignancies, including non-small-cell lung cancer (NSCLC) (65.2%), gastrointestinal cancer (11.8%), small-cell lung cancer (SCLC) (6.8%), breast cancer (4.3%), renal cell carcinoma (1.9%), and others (10 %). Most patients had oligometastases (87.6%) (13). 61.5% metastases were treated with SRT and 38.5% were treated with SRT + WBRT. The concurrent WBRT was defined according to Hunter et al. (14) as WBRT was completed within 1 month before or after SRT. In our study, 90% patients received concurrent WBRT and 10% patients received WBRT pre or post SRT.

**Table 1:** Patients characteristics.

**Characteristics**	**No./median** **(range)**	**Proportion (%)**
Sex		
Male	103	64
Female	58	36
Age (y)	61 (33–87)	
KPS		
≤ 70	90	55.9
>70	71	44.1
Histology		
NSCLC	105	65.2
SCLC	11	6.8
Breast	7	4.3
Renal	3	1.9
Gastrointestinal	19	11.8
Others	16	10.0
Primary tumor		
NSCLC	105	65.2
None NSCLC	56	34.8
Synchronous BM		
YES	53	32.9
NO	108	67.1
Extracranial metastases		
YES	91	56.5
NO	70	43.5
Number of treated lesions		
1	99	61.5
2	31	19.3
3	11	6.8
4	8	5.0
5	2	1.2
>5	10	6.2
Time from diagnosis to brain metastasis (M)	10 (0–300)	
System therapy cancer^*^	93	57.8
Total BM volume		
Per patient (cc)	8.79 (0.113–179.31)	
Prescription dose	27 (20–40)	
Fraction	5 (3–6)	
BED_10_	38.016 (16.6–84.375)	
Fraction	5 (3–10)	
SRT alone	99	61.5
SRT+WBRT	62	38.5
Controlled of primary tumor		
Controlled	92	57.1
Uncontrolled	69	42.9
GPA score		
0.5	9	5.6
1.0	22	13.7
1.5	40	24.9
2.0	30	18.6
2.5	30	18.6
3.0	20	12.4
3.5	9	5.6
4.0	1	0.6
RPA classification		
I	50	31.1
II	92	57.1
III	19	11.8

* *System therapy cancer: together with chemotherapy or targeted therapy*.

*KPS, Karnofsky performance status; BM, brain metastases; GPA, graded prognostic assessment; RPA, recursive partitioning analysis; SRT, stereotactic radiation therapy; WBRT, whole-brain radiotherapy*.

Patients treated with a median dose of 27Gy (20–40 Gy) were with 5–6 fractions. Ninety-one of ninety-nine patients who had neurological symptoms showed remission after SRT. Forty-nine (30%) patients suffered grade 1–2 toxicities with headache, dizziness, weakness, seizure, or edema. Four (2.5%) patients had serious cerebral necrosis and needed long-time treatment of bevacizumab (Tables 2, 3).

**Table 2:** Pre-SRT clinical symptoms and Post-SRT functional outcomes.

**Pre-SRT symptoms**	***n***	**Post- SRT**	***n***
Headache	40	Improved	33
Dizziness	32	Improved	26
Weakness	2	Improved	2
Dysarthria	11	Improved	6
Vomitting	18	Improved	18
Visual dysfunction	11	Improved	10
Epilepsy	3	Improved	1
Central ataxia	14	Improved	12
Cognitive dysfunction	4	Improved	2
Motor weakness	38	Improved	35
Hemiplegia	10	Improved	8
Hyperspasmia	7	Improved	5
Asymptomatic	63	New developed	15

*SRT, stereotactic radiation therapy*.

**Table 3:** Numbers of patients with 1–5 toxicities.

**Grade**	**Symptom**	***n***
1	Headache	15
	Dizziness	9
	Weakness	4
	Seizure	2
2	Edema	15
	Hemorrhage	2
	Seizure	2
3	Edema	9
	Hemorrhage	2
4	Edema	3
	Hemorrhage	1
	Cerebral necrosis	2
5	Hemorrhage	1
	Cerebral necrosis	2
Total		69

### Overall Survival

**Figure 1:**
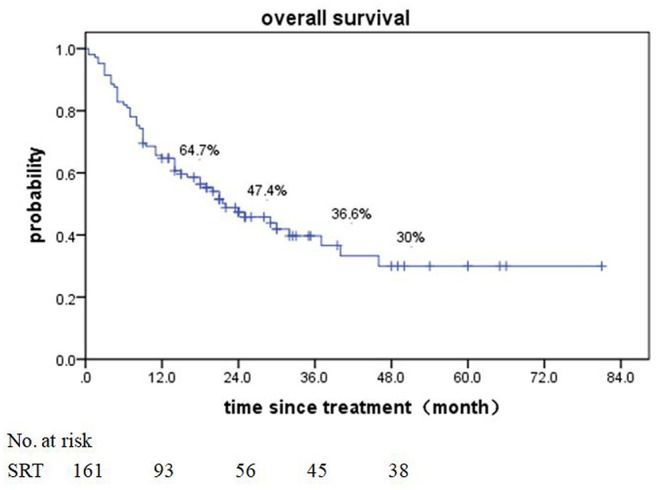
Overall survival of all 161 patients after SRT.

Of patients alive at last follow-up, the median follow-up was 48.5 months. The median overall survival (OS) after SRT was 19 months (range, 0.5–81 month) (Figure 1). The median BED was 39.15Gy (range, 16.8–84.375Gy). The median time from diagnosis to brain metastasis was 10M (range, 0–300 month). The median total lesion volume was 8.79 cc (range, 0.113–179.31cc).

The univariable analyses with Cox proportional hazards regression are shown in Table 4. The BED_10_-SRT (≥50 vs. <50, *p* = 0.05), a GPA of 2.5 significantly influenced OS (*P* = 0.003), the number of lesions treated (single lesion vs. multiple lesions, *p* = 0.005 and 1–3 lesions vs. more than 3 lesions, *p* < 0.001) significantly influenced OS. Targeted therapy also significantly influenced OS (24 months for targeted therapy vs. 13 months for no targeted therapy, *p* < 0.001). Combined with extracranial metastasis significantly influenced OS (13 months for with extracranial metastasis vs. 24 months for without, *p* < 0.016). Furthermore, controlled of extracranial disease also achieved significance (13.5 months for uncontrolled vs. 24 months for controlled, *p* = 0.011). RPA class I achieved a median OS of 31.5 months and class II achieved a median OS of 14 months (*p* = 0.026). Primary tumor type significantly also influenced OS (NSCLC achieved a median OS of 22 months and non-NSCLC achieved a median OS of 11 months, *P* = 0.005).

**Table 4:** Univariate analysis of predictors associated with OS.

**Variable**	**HR**	**95%CI**	*p*-value^*^
Age ( ≤ 61 vs. >61)	1.173	0.794–1.733	0.424
Gender (Male vs. Female)	1.481	0.977–2.246	0.064
Tumor volume (>8.79cc vs. ≤ 8.79cc)	1.332	0.855–2.074	0.296
KPS score (>70 vs. ≤ 70)	0.678	0.454–1.011	0.057
Synchronous BM (No vs. Yes)	0.811	0.538–1.224	0.319
Extracranial metastases (Yes vs. No)	1.640	1.096–2.453	**0.016**
BED_10_ (≥50 vs. <50)	0.547	0.299–0.999	**0.049**
Number of metastases			
Single vs. multiple	0.569	0.384–0.841	**0.005**
1–3 vs. >3	0.351	0.208–0.592	**<0.001**
GPA score (≥2.5vs. ≤ 2)	0.522	0.340–0.801	**0.003**
RPA classification			
Class I vs. II	0.592	0.373–0.940	**0.026**
Class I vs. III	0.729	0.367–1.450	0.368
Class II vs. III	1.208	0.650–2.245	0.549
Extracranial disease (Uncontrolled vs. Controlled)	1.672	1.127–2.481	**0.011**
Symptoms (YES vs. NO)	1.168	0.780–1.750	0.451
Treatment (SRT vs. SRT+WBRT)	0.930	0.627–1.380	0.717
Targeted therapy (YES vs. NO)	0.162	0.102–0.257	**<0.001**
Chemotherapy (YES vs. NO)	0.587	0.333–1.034	0.065
Tumor type (NSCLC vs. none NSCLC)	0.576	0.388–0.854	**0.006**

* *Univariable analysis with Cox proportional hazards regression; CI, confidence interval; Bold values indicate p < 0.05*.

SRT only, compared with concurrent WBRT, had no statistical significance (*p* = 0.717). There is no statistical significance for the time from diagnosis to brain metastasis (*p* = 0.319). Neurological symptoms before treatment had no significant influence (*p* = 0.451). There was a trend toward better survival rates for together with chemotherapy and higher KPS.

**Figure 2:**
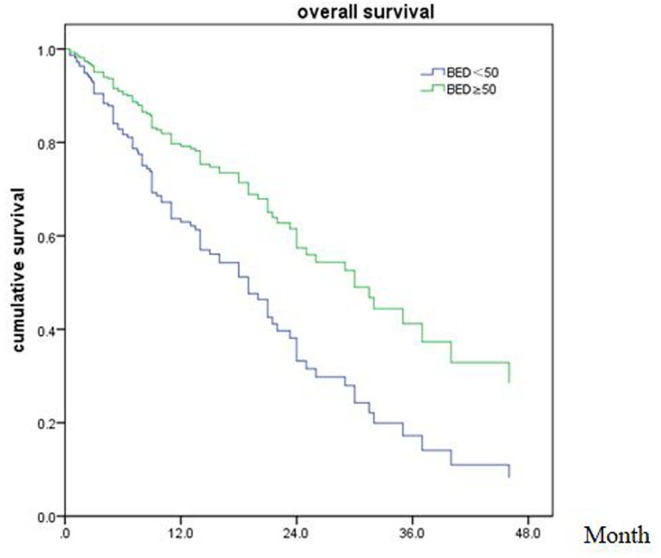
Overall survival of patients with BED ≥ 50 Gy and BED < 50 Gy (*p* = 0.027).

**Figure 3:**
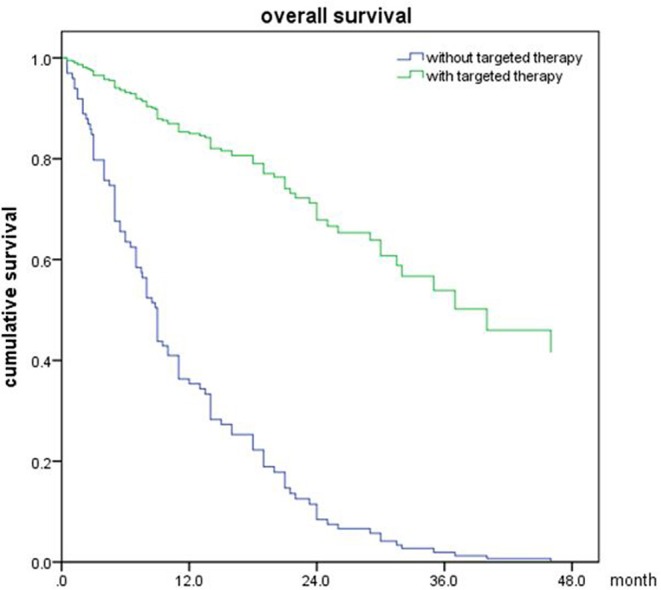
Overall survival of patients with targeted therapy and no targeted therapy (*p* < 0.001).

**Figure 4:**
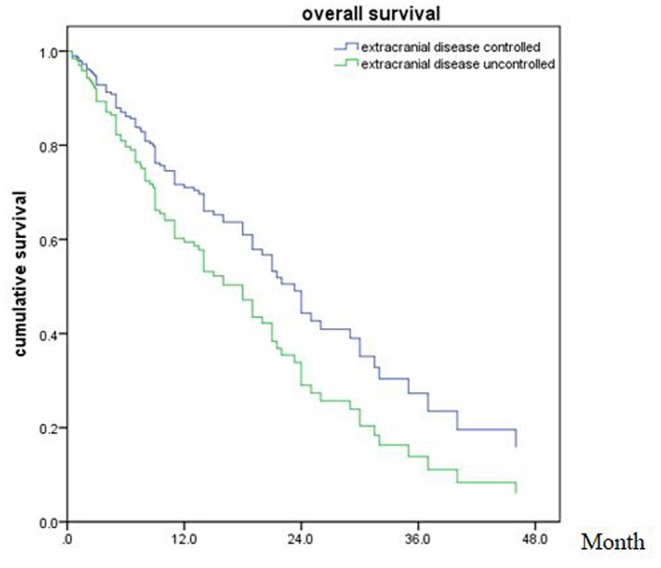
Overall survival of patients with extracranial disease controlled and uncontrolled (*p* = 0.039).

Of all 161 patients, multivariable analyses were shown in Table 5. BED_10_-SRT≥50Gy (*p* = 0.027), targeted therapy (*p* < 0.001) and controlled of extracranial disease (*p* = 0.039) were significant predictive factors (Figures 2–4).

**Table 5:** Multivariate analysis of predictors associated with OS.

**Variable**	**HR (95%CI)**	*P*-value^*^
Extracranial metastases		
YES	NA	0.509
NO	NA	
BED_10_		
≥50Gy	0.504 (0.275–0.924)	**0.027**
<50Gy	1 (ref)	
Number of metastases (1–3 vs. >3)		
Single	NA	0.279
Multiple	NA	
1–3	NA	0.529
>3	NA	
GPA score		
≥2.5	NA	0.883
≤ 2	NA	
Extracrinal disease		
Uncontrolled	1 (ref)	**0.039**
Controlled	0.658 (0.442–0.978)	
Targeted therapy		
YES	0.157 (0.098–0.250)	**<0.001**
NO	1 (ref)	
RPA classification		
Class I	NA	0.628
Class II	NA	
Tumor type		
NSCLC	NA	0.182
None NSCLC	NA	

* *Multivariate analysis using the Cox proportional hazards model; NA, Not Available; CI, confidence interval; Bold values indicate p < 0.05*.

**Figure 5:**
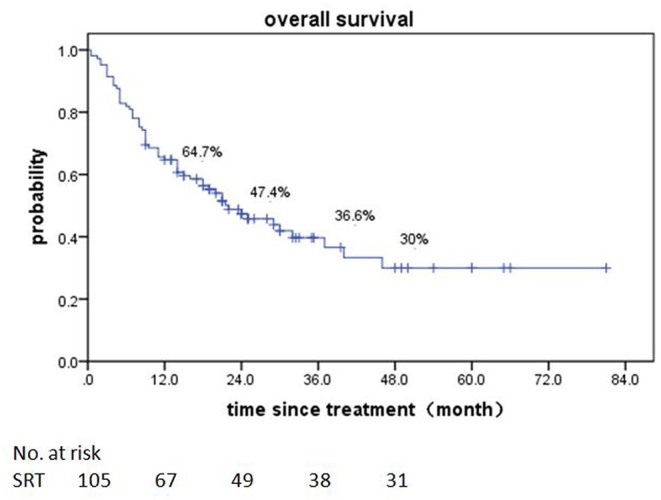
Overall survival of NSCLC patients after SRT.

**Figure 6:**
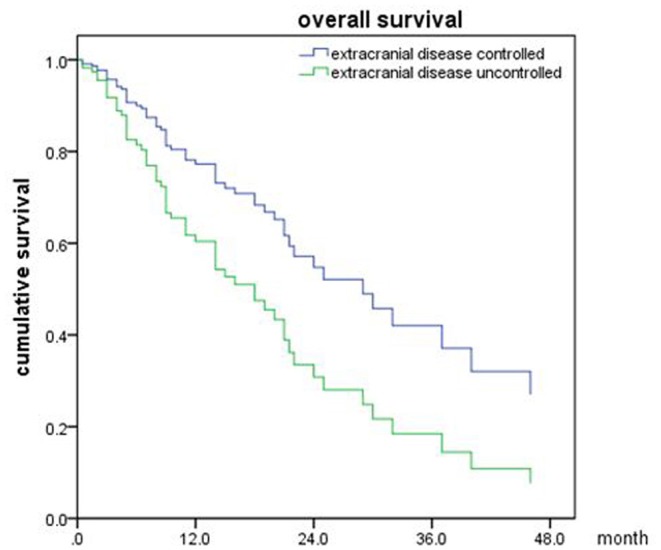
Overall survival of NSCLC patients with extracranial disease controlled and uncontrolled (*p* = 0.012).

**Figure 7:**
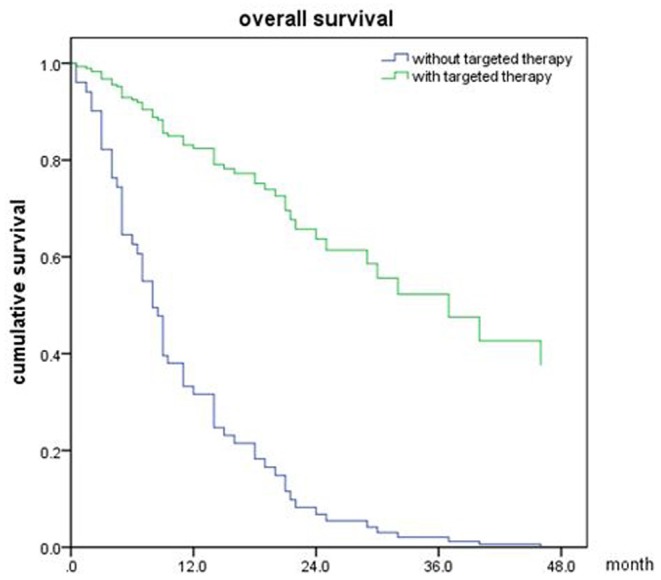
Overall survival of NSCLC patients with targeted therapy and no targeted therapy (*p* < 0.001).

In the meantime, the median OS after SRT was 22 months (range, 0.5–81 month) in NSCLC (Figure 5). The univariable analyses are shown in Table 6. In multivariable analysis, controlled of extracranial disease (*p* = 0.012) and targeted therapy (EGFR-TKI) (*p* < 0.001) were associated with improved OS (Table 7; Figures 6, 7).

**Table 6:** Univariate analysis of predictors associated with OS in NSCLC.

**Variable**	**HR**	**95%CI**	*p*-value^*^
Age ( ≤ 61 vs. >61)	1.193	0.794–1.733	0.502
Gender (Male vs. Female)	2.275	0.977–2.246	**0.009**
Tumor volume ( ≤ 8.79 cc vs. >8.79 cc)	1.332	0.855–2.074	0.296
KPS score (>70 vs. ≤ 70)	0.674	0.401–1.135	0.138
Synchronous BM (No vs. Yes)	0.729	0.435–1.222	0.231
Extracranial metastases (Yes vs. No)	1.603	0.956–2.689	0.074
BED_10_ (≥50 vs. <50)	0.547	0.299–0.999	0.090
Number of metastases (1–3 vs. >3)			
Single vs. multiple	0.665	0.397–1.113	0.121
1–3 vs. >3	0.386	0.165–0.908	**0.029**
GPA score (≥2.5 vs. ≤ 2)	0.628	0.370–1.067	0.085
RPA classification			
Class I vs. II	0.543	0.289–1.020	0.057
Class I vs. III	0.729	0.367–1.450	0.368
Class II vs. III	1.208	0.650–2.245	0.549
Extracranial disease (Uncontrolled vs. Controlled)	2.096	1.244–3.532	**0.005**
Symptoms (Yes vs. No)	1.214	0.723–2.039	0.463
Treatment (SRT vs. SRT+WBRT)	1.204	0.721–2.009	0.473
Targeted therapy (YES vs. NO)	0.161	0.088–0.294	**<0.001**
Chemotherapy (YES vs. NO)	0.587	0.333–1.034	0.083

* *Univariable analysis with Cox proportional hazards regression; CI, confidence interval; Bold values indicate p < 0.05*.

**Table 7:** Multivariate analysis of predictors associated with OS in NSCLC.

**Variable**	**HR (95%CI)**	*P*-value^*^
Number of metastases (1–3 vs. >3)		
1–3	NA	0.513
>3	NA	
Gender		
Male	NA	0.378
Female	NA	
Extracranial disease		
Uncontrolled	1 (ref)	**0.012**
Controlled	0.512 (0.303–0.865)	
Targeted therapy		
YES	0.168 (0.092–0.307)	**<0.001**
NO	1 (ref)	

* *Multivariate analysis using the Cox proportional hazards model; NA, Not Available; CI, confidence interval. Bold values indicate p < 0.05*.

## Discussion

In our study, we collected data of 161 eligible patients with BM in this study. The results showed that higher BED_10_-SRT, controlled of extracranial disease and targeted therapy were significant predictive factors.

WBRT is the common approach to the treatment for the patients with BM historically. Compared with WBRT, SRT alone or in combination with other modalities is generally used as the standard option for patients with BM especially oligometastasis, which leads to more clinical benefit and less toxicity.

For multiple brain metastases, WBRT was a standard choice for most cases over a long period of time. But the neurocognitive dysfunction cannot be ignored in longtime survival patients. Therefore, SRT has been more and more commonly used recently. Recent studies have shown that local treatments may minimize long-term neurocognitive dysfunction and improve quality of life (QOL) without compromising OS (15). Contrarily, Brown et al. (16) demonstrates that SRT alone may be associated with improved neurocognitive effects and quality of life despite the increased intracranial relapse rate. However, there have been no definitive conclusions whether treatment with SRT is as effective as that with WBRT or WBRT plus SRT in some specific number of brain metastases.

The prognostic factors related to better OS for patients with brain metastases have been studied in a large amount of clinical trials. Many prognostic scoring systems (17) have been proposed in the last 30 years to define the prognosis and better therapeutic option.

Study series showed that the factors of RPA class, GPA score, KPS, primary tumor category, extracranial diseases status, and number of brain lesions were variables associated with overall survival post-SRT (18, 19). A smaller trial (20) showed that combined WBRT and radiosurgery for patients with two to four brain metastases significantly improves local control of brain disease, but no improvement of survival.

Besides intracranial tumor burden, other clinical factors play an important role in treatment decisions. Performance status, age, extracranial metastases, and primary tumor control are all present in the GPA classification (21). As small samples and the paucity of data for SRT treating brain metastases, we analyzed the outcomes of patients with brain metastases treated with SRT with or without other treatments in different primary cancers.

Similarly, our study also explored extracranial diseases status (controlled vs. not controlled) were variables predicting OS. But we didn't find the relationship between OS and RPA, GPA, KPS score as well as the number of brain lesions. Possibly because patients who received SRT had better KPS and less neurological symptoms before treatment.

The most common primary cancers that metastasize to the brain are lung cancer, renal cancer, melanoma, colorectal cancer, and breast cancer. About 6% of those patients, brain metastases occur within 1 year of the diagnosis of the primary cancer (22). The failure of medical therapies in BM was well-known due to the lack of blood brain barrier (BBB) penetration. Fortunately the molecular targeted therapies have shown efficacy in the management of BM patients with activating mutations. Moreover, the target therapy was observed as a prognostic factor in BM patients, which can be effective for both intracranial as well as extracranial disease post-SRT.SRT alone had been widely accepted for treatment of oligo-brain metastases (1–4 brain metastases) In previous studies, the role of radiosurgery alone for patients with multiple brain metastases is still controversial. WBRT has classically been the standard treatment, while radiosurgery is commonly considered as a salvage therapy (23, 24).

Although the addition of WBRT improves intracranial control, it induces an increased risk of cognitive impairment without benefit in OS in the population of patients with brain metastasis, including patients with NSCLC (25–27).

A multi-institutional prospective observational study enrolled 1,194 patients with 1–10 brain metastases with an accumulated volume of all metastases <15 ml, treated with radiosurgery, showed that overall survival and toxicity did not differ between those with the 2–4 and 5–10 metastases groups (*p* = 0.78 median overall survival, 10.8 vs. 10.8 months, respectively) (28), which suggests that SRT-alone may be a reasonable treatment for patients with multiple brain metastases. The same result was also obtained in our study, WBRT was not independently associated with improved OS, no matter the primary tumor category or the number of brain lesions. However, the prospective randomized clinical trials are needed to evaluate the role of radiosurgery alone with omission of upfront WBRT in patients with multiple BM.

In the meanwhile, BED_10_ as an independent prognostic factor with OS was rarely reported before. In our study, an average prescription dose of 27Gy (20–40Gy) in 5–6 fractions was schemed, which was believed to be safe and effective dose for BM (29). Kumar et al. (30) reported that a higher total BED_10_ was statistically significant for improved local control (*p* = 0.04) with a threshold BED_10_≥48Gy associated with better local control for BM patients after surgical resection. We observed that BED_10_≥50Gy was associated with overall survival in the whole population of patients with BM, but not in BM from NSCLC. Previously the medical treatment was limited in BM patient because of blood-brain barrier. In the last decade, the targeted therapies of TKI had contributed to local control with concurrent radiotherapy for most NSCLC with activating mutations. Based on our data, we would recommend a higher BED_10_ for the patients lack of effective target drug or with relative radiation resistance primary tumor.

Some studies have reported nearly 10% of new NSCLC patients have brain metastases at diagnosis (31) and a further 25–40% patients will develop brain metastases during the course of disease (32). The NSCLC patients with EGFR mutation have a higher diagnosis rate of BM. The median OS is ~3–6 months or even less for patients without treatment (5, 33). A retrospective study showed SRT achieved better OS than WBRT or EGFR TKI alone (46 vs. 30 vs. 25 m respectively) in NSCLC patients with EGFR mutated (34).So cranial radiotherapy plays a critical role in patients with BM in NSCLC. In our research, we also find that EGFR-TKI (*p* < 0.001) and controlled of extracranial diseases (*p* = 0.012) were associated with improved OS in NSCLC patients.

Numerous studies have demonstrated that radiotherapy plus EGFR-TKIs led to more promising results than EGFR-TKIs or radiotherapy alone (35). SRT might be an optimal treatment for patients with EGFR mutations rather than WBRT. Thus, extracranial disease control is of the highly relevant with the OS of BM patients.

There are also some limitations in this study. Firstly, this is a retrospective study in a single institution, which included unrecognized biases and confounding factors. Secondly we could not collect much more details about the progression of the BM lesions during the long interval time of the follow-up, which caused the unmeasured intracranial PFS.

## Conclusion

As the development of radiotherapy, SRT adoption has dramatically improved the treatment outcomes compared with conventional fractionated radiotherapy for the BM patients. There was no difference in overall survival that has been observed between SRT alone compared to SRT plus WBRT in limited number of BM patients. The concurrent WBRT with SRT should be a cautious choice for selected patients. Our study confirmed that excellent extracranial disease controlled and BED_10_-SRT≥50Gy may predict a favorable prognosis in BM patients treated with SRT.

## Data Availability Statement

All datasets generated for this study are included in the article/supplementary material.

## Author Contributions

LG and SQ have made equal contribution to the study and provided the largest writing contribution of the manuscript. HZ was the main principal of this study. XZ, XJ, YC, ZJ, YS, FC, and FF have performed collection and organization of data. All authors approved it for publication.

### Conflict of Interest

The authors declare that the research was conducted in the absence of any commercial or financial relationships that could be construed as a potential conflict of interest.

## Abbreviations

SRT: Stereotactic Radiation Therapy
BM: brain metastases
WBRT: whole brain radiation therapy
EGFR: epidermal growth factor receptor
NSCLC: non–small-cell lung cancer
SCLC: small-cell lung cancer
CNS: Central Nervous System
SRS: stereotactic radiosurgery
OS: overall survival
GPA: Graded Prognostic Assessment
RPA: recursive partitioning analysis
BED_10_-SRT: biological effective dose of SRT
KPS: karnofsky performance score
TKI: Tyrosine Kinase Inhibitor
QOL: quality of life.
